# Milroy lecture 2024. Non-communicable versus communicable diseases: A paradigm unfit for the 21st century?

**DOI:** 10.1016/j.clinme.2025.100308

**Published:** 2025-04-02

**Authors:** Amitava Banerjee

**Affiliations:** Institute of Health Informatics, University College London, London, United Kingdom

**Keywords:** Non-communicable diseases, Communicable diseases, Clinical care, Pandemics, Covid-19

## Abstract

The dominant paradigm in clinical medicine, public health and global health has been to split clinical practice, research, education, training and funding into communicable versus non-communicable diseases. This dichotomy was borne out of the context of colonialism and increasingly, not least during the recent COVID-19 pandemic, evidence suggests that a much closer relationship between these ‘disease siloes’ would be beneficial, from risk factors to therapeutics. In this lecture, the synergies between communicable and non-communicable diseases are explored in order to inform future policy and practice.

## Introduction

Generations of medical students and public health professionals have been taught the ‘diseases of affluence’ versus the ‘diseases of poverty’ paradigm. The Global Burden of Disease Study (GBD), a driving force in comparison of cause-specific morbidity and mortality across countries for the last 30 years, refuted this concept in the 1990s.^1^ In fact, the early GBD was motivated by observations of discrepancies between international health sector need and spending.^2^

Non-communicable diseases (NCDs), including cardiovascular diseases and cancer, represent the largest burden of morbidity and mortality worldwide.^3^ Unfortunately, attention and resources have not been, and are not, proportionate to this disease burden at local, regional and global levels. A major contributor to this mismatch is that human, infrastructural and financial resources for NCDs are often in direct competition with communicable diseases (CDs).^4^

In one of the first scientific references to the term ‘non-communicable disease’, Gilliam noted in 1954:

‘It was not intended to imply that the large body of diseases now regarded as noncommunicable are necessarily so in fact .…. Communicability, however, is not fundamentally a concept of microbiology. It is a function of behaviour of disease in human populations and, as such, is an epidemiological concept.’^5^

However, from social determinants of health to pathophysiology, scientific thinking continues to be largely confined to CD versus NCD siloes,^6^ grossly neglecting these overlapping areas highlighted by Gilliam seven decades ago. The COVID-19 pandemic has recently challenged not only health systems, but also our paradigms of research and clinical practice. For example, the SARS-CoV-2 virus is considered to have had direct, indirect and long-term effects and its indirect impact on NCDs, including cardiovascular diseases and obesity, is substantial.^7^ Better integration of NCDs and CDs in practice and policy could improve science, public health and international security, but requires major shifts in thought and action.^8^

In this lecture, I explore the history and context of classification of CDs and NCDs. Using examples of synergies between these disease areas, I consider new ways of working across patients, professionals and policymakers.

### Historical context

To understand the origin of the CD/NCD distinction in both academic and clinical culture, it may be useful to investigate the history of the paradigms which have been most influential in medical practice over the last few centuries: medical specialisation, public health, humanitarian medicine, global health and tropical medicine. Medical specialisation is not a new concept. In ancient times, including Egyptian and Roman eras, there were practitioners who were particularly interested in certain organs or diseases. In the UK, medical specialisation really started to accelerate in the 18th century, especially with the advent of specialty professional societies in the late 19th and early 20th centuries.^9^ The earliest reference to ‘public health’ is credited to Fynes Moryson in 1617, meaning the overall health of the general population, but the concept only gradually became more widely known throughout Europe over the next two centuries. The term ‘humanitarian’ emerged around 1835, meaning ‘for the good of humanity’,^10^ and humanitarian medicine followed, encapsulating healthcare delivery in times of conflict and catastrophe. Post-Second World War, the formation of the United Nations in 1945 and the World Health Organization in 1948 led to the broader discourse around ‘global health’. In recent years, focus has shifted to the wider relationships between health and the environment, and term, ‘planetary health’, publicised in 2015 by a *Lancet* Commission.^11^

Of these dominant health and healthcare paradigms, none has been more instrumental to the division between CDs and NCDs as ‘tropical medicine’, which emerged in the late 19th century. The two oldest schools of tropical medicine in the UK, Liverpool and London, were founded in 1898 and 1899 by Sir Ronald Ross and Sir Patrick Manson respectively. Ross had made groundbreaking discoveries in malaria after serving in the Indian Medical Service for 25 years, and Manson uncovered the pathophysiology of filariasis after serving for many years in Taiwan (then Formosa), China and Hong Kong. It is perhaps important to note that these two schools were founded where there were hospitals for seamen, mostly returning from the UK’s colonies.^12^ Therefore, inevitably, tropical medicine and its focus on CDs was because experience and expertise in these diseases were needed for the benefit of UK citizens working in official capacity in colonies, ‘designed to facilitate exploitation of colonised places and people by colonisers’.^13^ It therefore follows that the distinction between CDs and NCDs, and the global focus on the former rather than the latter, has links, whether directly or indirectly, to colonialism. Hence, the argument against strict boundaries between CDs vs NCDs has a basis in epistemic injustice, as well as science. In India, once the ‘jewel of the British Empire’, these tensions are perhaps most apparent. Colonialism, including medicine at that time, was not simply to protect the British in India, it ‘encompassed the benevolence and hegemony of Western epistemology and therapeutic praxis’.^14^ The first medical officers arrived with the British East India Company’s first fleet in the 17th century, but it was not until 1946 that the health of the nation was properly reviewed by the Bhore Committee in terms of ‘Public Health, Medical Relief, Professional Education, Medical Research, and International Health’, that public health of Indians, including NCDs, was holistically considered.^15^

### What we already know

There is wide debate and disagreement with the siloed thinking of NCDs versus CDs.^16^ Consideration of global gaps in research and healthcare funding, implementation and translation of evidence-based interventions, and inadequate attention to social determinants of health, syndemics or health service research may help to move us forward in our thinking.

Sridhar and Batniji studied the four largest global health donors (the World Bank, Bill & Melinda Gates Foundation (BMGF), the US Government and the Global Fund to Fight HIV/AIDS, Tuberculosis and Malaria) in 2005. They highlighted major funding inequalities by disease compared with disease-specific disability-adjusted life-years: eg US $1,029.10 for HIV/AIDS to $3.21 for NCDs and signalled the pressing need for data transparency and standardisation.^4^ The ‘10–90 gap’ is a term used to describe the disparity between the amount of health research that is conducted in low- and middle-income countries and the amount of health research that is conducted in other parts of the world. It greatly underestimates the true deficiency in context-specific research in low- and middle-income countries,^17^ especially if we consider multimorbidity, which is an increasing concern to patients, professionals and policymakers.^18^

Looking at social determinants of health, whether ‘structural’ (eg racism and discrimination, education, socioeconomic status, the built environment) or ‘intermediary’ (eg the food environment, health literacy, stigma), these factors can and do influence both NCDs and CDs, often together. For example, healthcare and public health interventions for homeless individuals typically emphasise infectious diseases, such as blood-borne viruses and tuberculosis, even though there is high risk of cardiovascular diseases and NCDs, which are detectable and preventable.^19^

The syndemic concept describes ‘how infectious diseases cluster with pre-existing conditions, interact with them, and are driven by larger political, economic, and social factors’.^20^ In other words, NCDs, as well as CDs, can have significant impact during pandemics. For example, other than age, the major drivers of COVID-19 mortality during the recent pandemic have been underlying NCDs, including cancers, diabetes, cardiovascular diseases and chronic obstructive pulmonary disease.^21^

From a health service view, the non-COVID-19 effects of the pandemic have been across many, if not all, clinical specialties, yet NCDs tend to be manifestly excluded from any pandemic planning or response activities. Whether cancer-related outpatient activity, percutaneous coronary interventions and coronary bypass surgery or endoscopy procedures, there were steep reductions, starting in the early pandemic, which had not reached pre-pandemic levels even 2 years later.^22^ Each clinical specialty has generally developed its own strategy to tackle these challenges and backlogs, which seems a very inefficient way of working. CDs can have profound indirect effects for NCDs, and it is likely to be beneficial to not only think across individual NCDs, but also to look across both CDs and NCDs in research and care.

### What we are finding out

Long COVID has been defined as symptoms persisting beyond 3 months after initial SARS-CoV-2 infection, but underlying pathophysiology, biomarkers and effective treatments are still yet to be properly defined. In the UK alone, long COVID has been estimated to affect over 2 million individuals, comparable to many other NCDs, including cardiovascular disease and chronic obstructive pulmonary disease. To understand the mechanism of long COVID and how to treat it, new frameworks for research and care may be necessary such as learning health systems (linking science, evidence and care) and integrated care are necessary. The NIHR-funded STIMULATE-ICP programme is an example of such a research and care approach, involving both clinical trials as well as observational research using routine electronic health record data and qualitative methodology.^23^^,^^24^

Recovery of individuals with long COVID is associated with COVID-19 vaccination, particularly if it is before the index SARS-COV-2 infection and inversely related to severity of fatigue at presentation. Healthcare utilisation, whether in primary or secondary care, is significant and comparable with NCDs such as depression and heart failure.^25^ In post-COVID and long COVID states, electronic health record analyses suggest that COVID-19 vaccination is associated with both reduced mortality and hospitalisation, particularly in individuals with underlying cardiovascular disease.^26^ When does a post-viral condition become considered as a long-term condition, and although the triggering event is a CD, can the overlap with NCDs be incorporated into our thinking from aetiology to management? In the dedicated long COVID clinical service at University College London Hospitals NHS Trust, an integrated care approach combines CD and NCD lenses for management and rehabilitation of this new disease.^27^ Preliminary electronic health record analyses in England (which had specialist long COVID clinics), compared with Wales (which did not have long COVID clinics), suggest that such an integrated approach may reduce hospitalisation for people with long COVID at system level.^28^ Traditionally, large research cohorts have been collected, curated and analysed to study new diseases, one (or, at most, a few) at a time. Well-recorded and well-managed electronic health record data at scale have the potential to change this paradigm, whether in terms of health service (eg hospitalisation and primary care appointments) or patient-reported outcomes (eg quality of life, employment).

Over the winter months, vulnerable individuals (by age and by underlying NCDs) are recommended to have vaccinations against common viral illnesses, namely influenza, COVID-19 and more recently, respiratory syncytial virus to reduce the risk of hospitalisation and mortality from these infections. For influenza vaccination, trial evidence supports a clear reduction in major cardiovascular events, and large-scale electronic health record analyses support the same for COVID-19 vaccination.^26^^,^^29^ The same has not yet been shown for respiratory syncytial virus, but studies are underway. Vaccination rates are suboptimal for a number of reasons, but if their impact on NCD prevention is also considered, then there is even more reason to focus on increasing vaccination uptake for population health, in both primary and secondary prevention.

Traditionally, subtyping of diseases has been based on disease-specific cohorts with limited, disease-specific variables. For example, in heart failure, chronic kidney disease and type 2 diabetes, classification systems and management decisions in clinical practice rely on left ventricular ejection fraction, estimated glomerular filtration rate and glycosylated haemoglobin (HbA1c), respectively. If we take national-level, representative electronic health record data and modern data analytic methods such as machine learning, we may discover new subtypes of disease which may be more generalisable and applicable to clinical practice, offering new knowledge for underlying disease mechanisms and treatments. Moreover, there may be synergies and overlaps between diseases which are overlooked by such siloed subtyping. Advances in cardio-renal-metabolic medicine are proving.^30^, ^31^, ^32^ Unlike current disease subtypes, there are stronger associations with wide-ranging, long-term clinical outcomes, offering opportunities for true ‘precision medicine’. With unrestricted variables across NCDs and CDs which is possible in routine linked electronic health records at scale, and unsupervised machine learning, we may uncover new commonalities between CDs and NCDs and new avenues for disease management.

## Conclusions

A learning health system perspective can help to conceptualise NCDs as risk factors and outcomes for CDs and vice versa, which is a more accurate reflection of the reality. Science needs to better integrate CDs and NCDs, including the context of syndemics. Evidence continues to be more data-driven and patient-led, superseding opinion-driven and researcher-led paradigms. In this framework, care becomes more proactive and less reactive, and more geared towards prevention. Efforts to embrace multi-, inter-, trans-disciplinary research will be facilitated and enriched by closer working between clinical and research disciplines across CDs and NCDs, yet training and practice continue be along established specialty lines.^33^ Disease classification along historic, siloed lines should neither stand in our way to prepare and plan for pandemics and other shocks,^34^ nor to offer the best treatment and research for our patients. The 21st century offers many new opportunities to completely reimagine the fundamental paradigm of CDs vs NCDs in the best interests of science, evidence and care.

## CRediT authorship contribution statement

**Amitava Banerjee:** Conceptualization, Investigation, Methodology, Writing – original draft, Writing – review & editing.

## Declaration of competing interest

The authors declare that they have no known competing financial interests or personal relationships that could have appeared to influence the work reported in this paper.
